# Thermoresponsive Polymers of Poly(2-(*N*-alkylacrylamide)ethyl acetate)s

**DOI:** 10.3390/polym12112464

**Published:** 2020-10-24

**Authors:** Xue Liu, Yuwen Hou, Yimin Zhang, Wangqing Zhang

**Affiliations:** 1 Key Laboratory for Green Chemical Technology of Ministry of Education, School of Chemical Engineering and Technology, Tianjin University, Tianjin 300350, China; liuxue435@tju.edu.cn; 2 Key Laboratory of Functional Polymer Materials of the Ministry of Education, Institute of Polymer Chemistry, College of Chemistry, Nankai University, Tianjin 300071, China; 1120180337@mail.nankai.edu.cn

**Keywords:** thermoresponsive, cloud point temperature, RAFT polymerization, block copolymers

## Abstract

Thermoresponsive poly(2-(*N*-alkylacrylamide) ethyl acetate)s with different *N*-alkyl groups, including poly(2-(*N*-methylacrylamide) ethyl acetate) (PNMAAEA), poly(2-(*N*-ethylacrylamide) ethyl acetate) (PNEAAEA), and poly(2-(*N*-propylacrylamide) ethyl acetate) (PNPAAEA), as well as poly(*N*-acetoxylethylacrylamide) (PNAEAA), were synthesized by solution RAFT polymerization. Unexpectedly, it was found that there are induction periods in the RAFT polymerization of these monomers, and the induction time correlates with the length of the *N*-alkyl groups in the monomers and follows the order of NAEAA < NMAAEA < NEAAEA < NPAAEA. The solubility of poly(2-(*N*-alkylacrylamide) ethyl acetate)s in water is also firmly dependent on the length of the *N*-alkyl groups. PNPAAEA including the largest *N*-propyl group is insoluble in water, whereas PNMAAEA and PNEAAEA are thermoresponsive in water and undergo the reversible soluble-to-insoluble transition at a critical solution temperature. The cloud point temperature (*T*_cp_) of the thermoresponsive polymers is in the order of PNEAAEA < PNAEAA < PNMAAEA. The parameters affecting the *T*_cp_ of thermoresponsive polymers, e.g., degree of polymerization (DP), polymer concentration, salt, urea, and phenol, are investigated. Thermoresponsive PNMAAEA-*b*-PNEAAEA block copolymer and PNMAAEA-*co*-PNEAAEA random copolymers with different PNMAAEA and/or PNEAAEA fractions are synthesized, and their thermoresponse is checked.

## 1. Introduction

Stimuli-responsive polymers have gained great attention due to their promising applications in drug delivery, separations, filtration, smart surfaces, and regulating enzyme activity [1,2,3,4,5]. The stimuli can be classified as chemical stimuli, e.g., pH [6,7,8,9,10,11,12,13,14] and chemical agents [15,16,17], and physical stimuli including temperature [8,10,12,18,19,20,21,22,23,24,25], electric or magnetic fields [26,27,28], and mechanical stress [29,30,31]. In all these stimuli, temperature is one of the most commonly used because of its easy control [19,32,33]. Five kinds of thermoresponsive polymers owing to a lower critical solution temperature (LCST) in water, e.g., poly(meth)acrylamides [19,34], poly(aminoethyl methacrylate)s [11,23,35,36,37], poly[oligo(ethylene glycol) (meth)acrylate]s [38,39,40,41,42], poly(2-alkyl-2-oxazoline)s [18,43,44,45], and poly(vinyl methyl ether)s [20,46], are the most popularly studied. These LCST-type polymers are soluble in water below the cloud point temperature *(T*_cp_) on account of hydrogen bonding between polymer chains and the surrounding water molecules. At a temperature above *T*_cp_, hydrogen bonding between polymer chains and water is destroyed and intra- and inter-molecular hydrogen bonding/hydrophobic interactions within the polymer chains dominate, which results in polymer insolubility accompanied by visible turbidity. This process of temperature-triggered solubility change is generally reversible, and the dehydrated polymer can re-dissolve in water to its initial state when the polymer solution cools down. Sometimes, a hysteresis in the dissolution process is generated, attributed to the formation of intra- and inter-chain hydrogen bonding within polymer chains [47]. For example, poly(*N*-isopropylacrylamide) (PNIPAM) with an LCST of 32 °C and *N*-isopropylacrylamide copolymers have slight hysteresis, generally within 2–6 °C, in the dissolution process [48,49,50]. However, poly(*N*,*N*-diethylacrylamide) (PDEAM) with an LCST of 35 °C exhibits no hysteresis, due to a lack of hydrogen bond donors [51,52,53].

The cloud point temperature, *T*_cp_, is possibly the most important parameter for thermoresponsive polymers. Thermoresponsive polymers in the aqueous solution with a suitable *T*_cp_ can be achieved by designing new monomers [54,55,56], by copolymerization of monomers with different hydrophilicity [21,50,57,58,59] and/or by addition of salts [22,23,60,61,62,63,64], urea [24,65,66], phenol [67,68], and surfactants [69,70]. Though a number of thermoresponsive polymers have been reported, thermoresponsive polymers having a *T*_cp_ very close to body temperature are rare [55,56]. Therefore, synthesis of such thermoresponsive polymers is of primary interest.

In this study, new thermoresponsive polymers of poly(2-(*N*-alkylacrylamide) ethyl acetate)s and their block and random copolymers are synthesized by solution RAFT polymerization. It is found that the *T*_cp_ of thermoresponsive poly(2-(*N*-alkylacrylamide) ethyl acetate)s firmly correlates to the *N*-alkyl group, and a thermoresponsive polymer with a *T*_cp_ very close to human body temperature is synthesized. The parameters affecting *T*_cp_, including the degree of polymerization (DP), polymer concentration, salt, urea, and phenol, are investigated.

## 2. Experimental

### 2.1. Materials

2-(Methylamino)ethanol (99%, Innochem, Beijing, China), 2-(ethylamino)ethanol (98%, Alfa Aesar, Shanghai, China), and 2-(propylamino)ethanol (98%, Sigma-Aldrich, Shanghai, China) were used as received. Acryloyl chloride (99%) was purchased from Heowns (Tianjin, China) and used directly. Trimethylchlorosilane (TMSCl, 99%), triethylamine (TEA, 99%), acetic anhydride, ethyl acetate, light petroleum, methanol, ethanol, and *N*,*N*-dimethylformamide (DMF) were of analytical grade and were purchased from Tianjin Chemical Company (Tianjin, China). Dichloromethane (DCM, Tianjin, China) of analytical grade purchased from Tianjin Chemical Company was used after distillation. 2,2’-Azobis(2-methylpropionitrile) (AIBN, >99%, Tianjin Ruijinte Chemical Reagent Co., Ltd., Tianjin, China) was used before recrystallization in anhydrous ethanol. Ethyl cyanovaleric trithiocarbonate (ECT) (Scheme S1) used as a chain transfer agent (CTA) was synthesized as discussed elsewhere [71]. Other chemicals were of analytical grade and were used as obtained. Deionized water was used in this study to prepare the aqueous solution of thermoresponsive polymers.

### 2.2. Synthesis of Monomers

#### 2.2.1. Synthesis of 2-(*N*-methylacrylamide) ethyl acetate (NMAAEA)

**Scheme 1:**
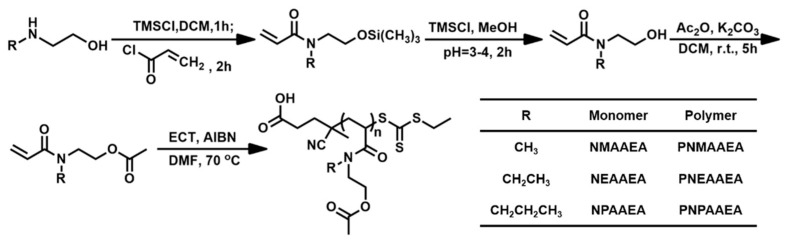
Synthesis of 2-(*N*-alkylacrylamide) ethyl acetate)s and poly(2-(*N*-alkylacrylamide) ethyl acetate)s.

The synthesis of 2-(*N*-alkylacrylamide) ethyl acetate monomers is shown in Scheme 1. Herein, the typical synthesis of 2-(*N*-methylacrylamide) ethyl acetate (NMAAEA) is introduced. 2-(Methylamino)ethanol (5.00 g, 66.56 mmol), anhydrous dichloromethane (70 mL), and TEA (14.82 g, 146.45 mmol) were added into a flask. The mixture was magnetically stirred under argon atmosphere, and then TMSCl (7.59 g, 69.90 mmol) was added dropwise to the reaction solution under 0 °C. After 1 h, acryloyl chloride (6.03 g, 66.56 mmol) was added dropwise to the flask. After being kept at room temperature for 2 h, the mixture was washed with water (3 × 90 mL). The aqueous layer was poured out and the organic layer was collected and then dried with anhydrous MgSO_4_. After MgSO_4_ was filtered out, the organic solvent was removed under reduced pressure to obtain *N*-methyl-*N*-2-(1-trimethylsiloxy) ethylacrylamide. The obtained *N*-methyl-*N*-2-(1-trimethylsiloxy) ethylacrylamide and anhydrous methanol (70 mL) were added into another flask with a magnetic bar, and then TMSCl (about 0.44 mL) was added dropwise to the flask under argon atmosphere at room temperature with pH at 3–4. After 2 h, the reaction mixture was concentrated under reduced pressure and the crude product was purified with column chromatography using ethyl acetate/methanol to afford a colorless product of *N*-methyl-*N*-2-(1-hydroxy) ethylacrylamide with 52% yield (4.50 g). Into a flask with a magnetic stirrer, *N*-methyl-*N*-2-(1-hydroxy) ethylacrylamide (4.00 g, 30.97 mmol), DCM (56 mL), potassium carbonate (6.85 g, 49.55 mmol), and acetic anhydride (3.48 g, 34.06 mmol) were added. The reaction mixture was filtered after it was stirred for 5 h at ambient temperature. The filtrate was concentrated by rotary evaporation under reduced pressure and the crude product was purified with column chromatography using ethyl acetate/light petroleum to obtain a colorless NMAAEA product with a yield of 78.5% (4.16 g). ^1^H NMR (400 MHz, CDCl_3_, ppm) *δ* 6.38–6.48 (m, 1H, CH_2_=CH–), 6.09–6.15 (m, 1H, CH_2_=CH–), 5.49–5.52 (m, 1H, CH_2_=CH–), 4.01–4.06 (m, 2H, –CH_2_–CH_2_–O–), 3.46–3.51 (m, 2H, –N–CH_2_–), 2.96 (s, 1.5H, –N–CH_3_), 2.84 (s, 1.5H, –N–CH_3_), 1.84 (s, 3H, –C–CH_3_). See the ^1^H NMR spectra in Figure S1. As shown by the ^1^H NMR spectra, imine-enamine tautomerism in the NMAAEA occurs similarly to those described elsewhere [72].

#### 2.2.2. Synthesis of 2-(*N*-ethylacrylamide) ethyl acetate (NEAAEA)

The synthesis of NEAAEA was similar to that of NMAAEA except for replacing 2-(methylamino)ethanol with 2-(ethylamino)ethanol. ^1^H NMR (400 MHz, CDCl_3_, ppm) *δ* 6.40–6.51 (m, 1H, CH_2_=CH–), 6.18–6.22 (m, 1H, CH_2_=CH–), 5.54–5.57 (m, 1H, CH_2_=CH–), 4.04–4.11 (m, 2H, –CH_2_–CH_2_–O–), 3.48–3.51 (m, 2H, –N–CH_2_–CH_3_), 3.30–3.36 (m, 2H, –N–CH_2_–CH_2_–), 1.90 (s, 3H, –C–CH_3_), 1.05–1.09 (t, 3H, –CH_2_–CH_3_). See the ^1^H NMR spectra in Figure S1.

#### 2.2.3. Synthesis of 2-(*N*-propylacrylamide) ethyl acetate (NPAAEA)

NPAAEA was obtained similarly to the synthesis of NMAAEA. ^1^H NMR (400 MHz, CDCl_3_, ppm) *δ* 6.35–6.49 (m, 1H, CH_2_=CH–), 6.12–6.17 (m, 1H, CH_2_=CH–), 5.48–5.51 (m, 1H, CH_2_=CH–), 3.99–4.06 (m, 2H, –CH_2_–CH_2_–O–), 3.44–3.47 (m, 2H, –N–CH_2_–CH_2_–O), 3.16–3.21 (m, 2H, –N–CH_2_–CH_2_–CH_3_), 1.85 (m, 3H, –C–CH_3_), 1.39–1.49 (m, 2H, –CH_2_–CH_3_), 0.72–0.76 (t, 3H, –CH_2_–CH_3_). See the ^1^H NMR spectra in Figure S1.

### 2.3. Synthesis of Poly(2-(N-alkylacrylamide) ethyl acetate)s by RAFT Polymerization

Poly(2-(*N*-alkylacrylamide) ethyl acetate)s were synthesized by solution RAFT polymerization using AIBN as initiator and ECT as CTA. Herein, the typical synthesis of poly(2-(*N*-methylacrylamide) ethyl acetate) (PNMAAEA) under [NMAAEA]_0:_[ECT]_0_:[AIBN]_0_ = 1000:5:1 is introduced. Into a Schlenk flask with a magnetic bar, NMAAEA (1.71 g, 9.99 mmol), ECT (13.16 mg, 0.050 mmol), the internal standard 1,3,5-trioxane (89.98 mg, 1.00 mmol), and AIBN (1.64 mg, 0.010 mmol) dissolved in DMF (3.42 g) were added. After degassing by three cycles of freeze-pump-thaw to remove any oxygen from the solution, polymerization was conducted at 70 °C. After a prescribed time, polymerization was quenched by putting the flask in ice water. The monomer conversion was detected by NMR analysis by comparing the integral areas at *δ* = 5.49–5.52 ppm (C=C–H) assigned to the monomer with those at *δ* = 5.15 ppm assigned to the internal standard 1,3,5-trioxane. After precipitation in ice diethyl ether, the synthesized PNMAAEA was dried in vacuum at ambient temperature overnight.

PNEAAEA and PNPAAEA were synthesized also by solution RAFT polymerization similar to PNMAAEA. However, for PNPAAEA, it was precipitated in ice *n*-hexane. The detailed synthesis of polymers is shown in Table S1.

### 2.4. Synthesis of Thermoresponsive Block Copolymers

The block copolymer PNMAAEA_198_-*b*-PNEAAEA_80_, where the subscript represents the theoretical DP herein and subsequently, calculated by assuming RAFT polymerization with quantitative chain transfer agent efficiency, was prepared by sequential RAFT polymerization using PNMAAEA_198_ as macromolecular CTA (macro-CTA) under [NEAAEA]_0_:[PNMAAEA_198_]_0_:[AIBN]_0_ = 420:5:1. Into a Schlenk flask with a magnetic bar, PNMAAEA_198_ (0.110 g, 0.00322 mmol), NEAAEA (50.00 mg, 0.270 mmol), the internal standard 1,3,5-trioxane (2.43 mg, 0.027 mmol), and AIBN (0.11 mg, 0.00060 mmol) dissolved in DMF (0.32 g) were added. The flask content was degassed, and polymerization was run at 70 °C for 6 h with 95.8% NEAAEA conversion. The synthesized PNMAAEA_198_-*b*-PNEAAEA_80_ was precipitated in iced diethyl ether and dried under vacuum at room temperature overnight.

### 2.5. Synthesis of Thermoresponsive Random Copolymers

The random copolymer PNMAAEA-*co*-PNEAAEA was synthesized by solution RAFT polymerization. Herein, the typical synthesis of PNMAAEA_100_-*co*-PNEAAEA_100_ under [NMAAEA]_0_:[NEAAEA]_0_:[ECT]_0_:[AIBN]_0_ = 500:500:5:1 is presented. Into a Schlenk flask with a magnetic bar, NMAAEA (0.30 g, 1.75 mmol), NEAAEA (0.32 g, 1.75 mmol), ECT (4.62 mg, 0.0175 mmol), the internal standard 1,3,5-trioxane (31.57 mg, 0.35 mmol), and AIBN (0.58 mg, 0.0035 mmol) dissolved in DMF (1.24 g) were added. After degassing the flask content, the Schlenk flask was immersed in a preheated oil bath at 70 °C for 24 h, and then quenched in iced water. The monomer conversions for both NMAAEA and NEAAEA determined by ^1^H NMR were 100%. The synthesized PNMAAEA_100_-*co*-PNEAAEA_100_ was purified by three precipitation–filtration cycles in cold diethyl ether and was dried under vacuum at room temperature overnight.

### 2.6. Characterization

The ^1^H NMR analysis was accomplished on a Bruker Avance III 400 MHz NMR spectrometer using CDCl_3_ with the proton signal at *δ* = 7.26 ppm as solvent. The polymer molecular weight (*M*_w_) and polydispersity (*Đ*, *Đ* = *M*_w_/*M*_n_) were determined by gel permeation chromatography (GPC) equipped with a Waters 600E GPC system at 50 °C employing THF as eluent, in which a series of poly(methyl methacrylate)s (PMMAs) with narrow-polydispersity molecular weight were used as calibration standards. Turbidity analysis was monitored by a Varian 100 UV-vis spectrophotometer equipped with a thermo-regulator (±0.1 °C) at 500 nm with a heating/cooling rate of 1.0 °C min^−1^, in which the transmittance was normalized with deionized water and samples were tested three times in order to obtain a standard deviation analysis as error. *T*_cp_ was determined by a 50% change in the transmittance. The differential scanning calorimetry (DSC) analysis was performed on a TA DSC Q100 differential scanning calorimeter under nitrogen atmosphere at 0.15 MPa. All the samples were initially heated to 160 °C at a heating rate of 10 °C min^−^^1^, then cooled to –60 °C and kept for 5 min, and finally heated to 160 °C at a heating rate of 10 °C min^−^^1^. Dynamic light scattering (DLS) analysis was carried out on a NanoBrook Omni laser light scattering spectrometer (Brookhaven, GA, USA) at the wavelength of 659 nm at 90° angle.

## 3. Results and Discussion

### 3.1. Synthesis of Monomers and Poly(2-(N-alkylacrylamide) ethyl acetate)s

The synthesis of 2-(*N*-alkylacrylamide) ethyl acetate monomers is shown in Scheme 1. The 2-(*N*-alkylacrylamide) ethyl acetate monomers were synthesized initially by amidation between acryloyl chloride and 2-(alkylamino)ethanol, in which the hydroxyl group was protected by TMSCl, to form *N*-alkyl-*N*-2-(1-hydroxy)ethylacrylamide and then followed by an esterification between *N*-alkyl-*N*-2-(1-hydroxy)ethylacrylamide and acetic anhydride. All the three monomers were confirmed by NMR analysis (Figure S1).

**Figure 1:**
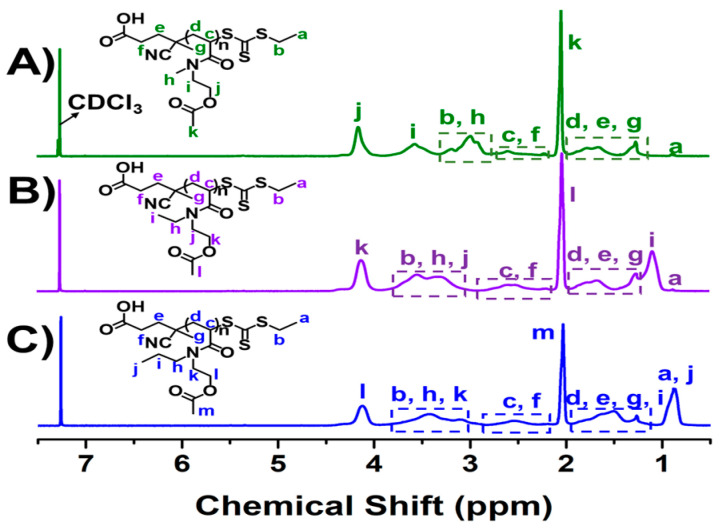
^1^H NMR spectra of PNMAAEA_198_ (**A**), PNEAAEA_194_ (**B**), and PNPAAEA_190_ (**C**) in CDCl_3_.

**Figure 2:**
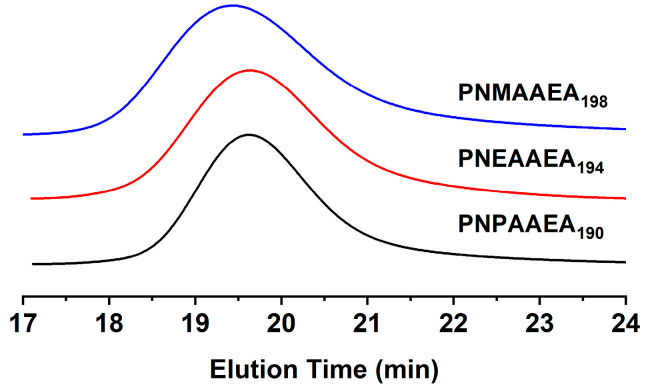
GPC traces of PNMAAEA_198_, PNEAAEA_194_, and PNPAAEA_190_.

Initially, three polymers with similar DP, e.g., PNMAAEA_198_, PNEAAEA_194_, and PNPAAEA_190_, were synthesized under [monomer]_0_:[ECT]_0_:[AIBN]_0_ = 1000:5:1 at an almost 100% monomer conversion to check the polymerization conditions. Figure 1 and Figure 2 show the ^1^H NMR spectra and GPC traces of the three polymers. For PNMAAEA_198_ and PNEAAEA_194_, the signal of the CTA moieties at about the chemical shift of 0.83 ppm can be discerned, and therefore, the molecular weight *M*_n,NMR_ can be calculated by comparing the chemical shifts δ at 0.83 and 3.84–4.48 ppm. As summarized in Table S1, for PNMAAEA_198_, its *M*_n,NMR_ is very close to the theoretical molecular weight *M*_n,th_ determined by Equation (S1). This indicates high introduction of the CTA moieties in the polymer chain end in the RAFT polymerization. For PNEAAEA_194_, *M*_n,NMR_ is lower than *M*_n,th_, indicating that the chain transfer agent efficiency in the synthesis of PNEAAEA_194_ is not as high as that in the synthesis of PNMAAEA_198_. As indicated by Scheme 1, NEAAEA and NMAAEA have the same chemical composition, and a similar chain transfer agent efficiency in the RAFT polymerization was expected. However, the fact is much different and the exact reason needs further study. For PNPAAEA_190_, it is difficult to discern the δ signal at 0.83 ppm assigned to the CTA moieties, and therefore, *M*_n,NMR_ is not calculated. By GPC analysis, the molecular weight *M*_n,GPC_ and *Đ* were obtained. It is found that all three polymers have a narrow molecular weight distribution, as indicated by *Đ* below 1.2, and *M*_n,GPC_ is lower than *M*_n,th_. The underestimation of *M*_n,GPC_ is possibly ascribed to the interaction between *N*-containing polymers and GPC columns, which is widely reported elsewhere [25,71]. Besides, the underestimation of *M*_n,GPC_ is also due to the poly(methyl methacrylate) calibration standards used in GPC analysis.

**Figure 3:**
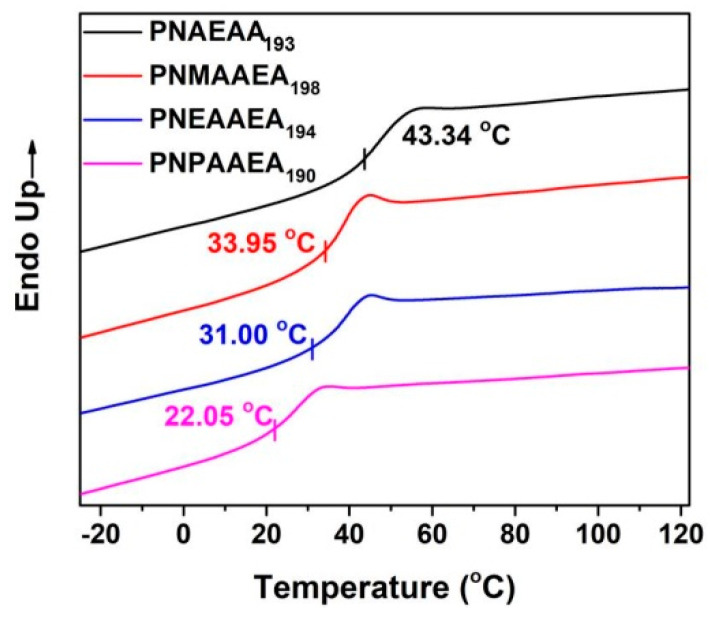
DSC thermograms of PNAEAA_193_, PNMAAEA_198_, PNEAAEA_194_, and PNPAAEA_190_.

Figure 3 shows that the *N*-alkyl group in poly(2-(*N*-alkylacrylamide) ethyl acetate)s firmly correlates to the glass transition temperature (*T*_g_), which is determined at the middle point in the step transition, as indicated by the intersection point in the DSC thermograms. The bigger *N*-alkyl group leads to a lower *T*_g_. That is, *T*_g_ is in the order of PNMAAEA_198_ > PNEAAEA_194_ > PNPAAEA_190_. For comparison, poly(*N*-acetoxylethylacrylamide) (PNAEAA_193_) with a similar structure but with the *N*-alkyl group replaced with a H atom was synthesized as reported previously [73]. It is found that PNAEAA_193_ has a much higher *T*_g_ of 43.34 °C than the other three homopolymers. It is thought that due to the *N*-alkyl group being replaced by H, there exists hydrogen bonding or strong intermolecular interaction in the PNAEAA chains, and therefore, PNAEAA has a higher *T*_g_.

**Figure 4:**
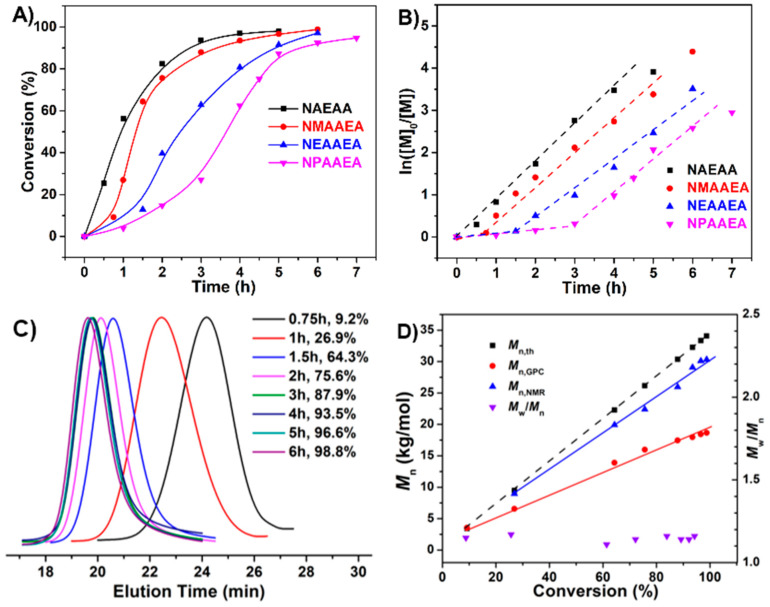
The time-dependent monomer conversion (**A**), the ln([M]_0_/[M])–time plots of the RAFT polymerization (**B**), the GPC traces (**C**), and the molecular weight and *Đ* (*M*_w_/*M*_n_) value of PNMAAEA at different conversions (**D**). Polymerization conditions: [NMAAEA]_0_ :[RAFT]_0_ :[AIBN]_0_ = 1000:5:1, 70 °C, solid content: 33.3%, solvent: DMF.

The kinetics of RAFT polymerization under a constant ratio of [Monomer]_0_:[CTA]_0_:[AIBN]_0_ = 1000:5:1 is checked. As summarized in Figure 4, for *N*-acetoxylethylacrylamide (NAEAA), there is almost no induction period in RAFT polymerization, and with the *N*-alkyl group becoming larger, the induction period increases from 30 to 150 min. RAFT polymerization undergoing an induction period is widely observed in solution RAFT formulation [72,74,75,76,77], and it is ascribed to the slow fragmentation of the initiator to produce leaving group radicals, the slow re-initiation by the expelled radicals, the increased stability of the intermediate radicals, and/or the low efficiency of chain transfer agent in capturing radicals. After the induction periods, all RAFT polymerizations follow pseudo-first-order kinetics with close to the same polymerization rates as indicated by the linear ln([M]_0_/[M])–time plots. As clearly shown in Figure 4B, the induction periods increase with the increase in the length of the *N*-alkyl substituents (carbon number) in the 2-(*N*-alkylacrylamide) ethyl acetate monomers in the following order: NAEAA < NMAAEA < NEAAEA < NPAAEA. This unexpected effect indicates that the structure of the *N*-alkyl group in the monomers plays a significant role in the processes leading to the induction periods, and investigating this phenomenon in more detail will be the subject of future investigations. The synthesized PNMAAEA, PNEAAEA, and PNPAAEA were characterized by NMR (herein, the ^1^H NMR spectra are not shown) and GPC (Figure 4C and Figure S2). As summarized in Figure 4D, all polymers have a narrow molecular weight distribution, as indicated by *Đ* below 1.17. *M*_n,GPC_ and *M*_n,NMR_ of the synthesized polymers linearly increase with monomer conversion, *M*_n,NMR_ is very close to *M*_n,th_, and *M*_n,GPC_ is smaller than *M*_n,th_. The underestimation of *M*_n,GPC_ is as discussed above.

### 3.2. Thermoresponse of Poly(2-(N-alkylacrylamide) ethyl acetate)s

In this section, the thermoresponse of the aqueous solution of poly(2-(*N*-alkylacrylamide) ethyl acetate)s, as well as PNAEAA, is investigated. It is found that PNPAAEA_190_ is insoluble in water, and therefore, the further investigation is focused on PNAEAA_193_, PNMAAEA_198_, and PNEAAEA_194_.

**Figure 5:**
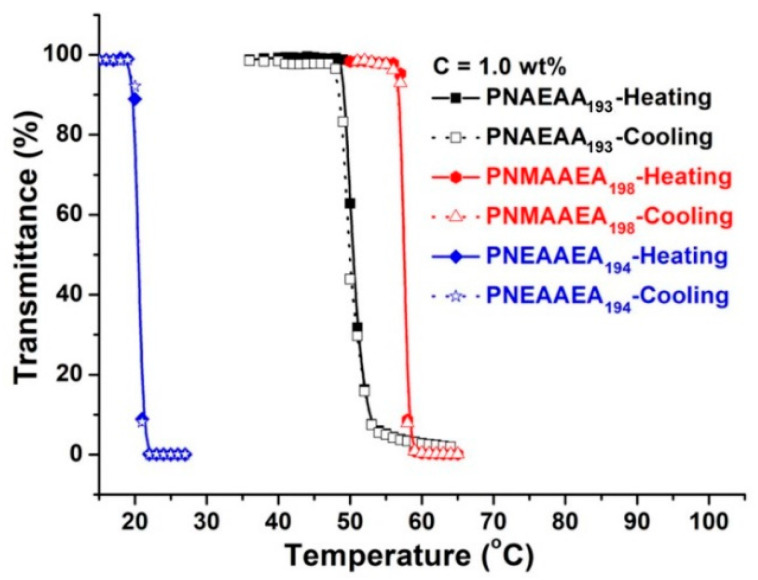
Temperature-dependent transmittance of 1.0 wt % aqueous solutions of PNAEAA_193_, PNMAAEA_198_, and PNEAAEA_194_.

As shown in Figure 5, PNAEAA_193_, PNMAAEA_198_, and PNEAAEA_194_ underwent a soluble-to-insoluble transition when the temperature increased above *T*_cp_ and a reversible insoluble-to-soluble transition when the temperature decreased below *T*_cp_. Note that, herein, *T*_cp_ is determined at a 50% change in the transmittance, where the temperature increases from below *T*_cp_ to above *T*_cp_. Readers can also refer to the *T*_cp_ determined at the inflection points in the heating process (Table S2). From Figure 5, three conclusions are made. First, *T*_cp_ firmly correlates to the *N*-alkyl group in poly(2-(*N*-alkylacrylamide) ethyl acetate)s. In the case of the H atom, PNAEAA_193_ has a moderate *T*_cp_ of 50.4 °C; in the case of the *N*-ethyl group, PNEAAEA_194_ has the lowest *T*_cp_ of 20.5 °C; and in the case of the *N*-methyl group, PNMAAEA_198_ has the highest *T*_cp_ of 57.5 °C. It was expected that PNAEAA_193_ should have the highest *T*_cp_ as the *N*–H group is more hydrophilic than either the *N*-methyl or *N*-ethyl group. However, the fact is inconsistent with the expectation. It is thought that intermolecular hydrogen bonding in PNAEAA chains depresses the hydrogen bonding between PNAEAA and water, and therefore decreases the *T*_cp_ of PNAEAA. Second, the phase transition takes place within a narrow temperature window of 1–3 °C. Third, there is almost no hysteresis in the cooling process, indicating a fully reversible solubility transition. For PNMAAEA_198_ and PNEAAEA_194_, this is reasonable, as the absence of a proton donor in PNMAAEA and PNEAAEA, leading to the weak intermolecular interaction, accounts for the reversible solubility transition without hysteresis. For PNAEAA, it seems a little surprising due to the strong intra- and inter-molecular hydrogen bonding ascribed to the *N–*H group in the polymer chains. Herein, it is thought that the inter- and/or intra- molecular hydrogen bonding in PNAEAA chains dominates over the intermolecular hydrogen bonding between the polymer chains and water molecules, which therefore leads to no or very slight hysteresis in the cooling process.

**Figure 6:**
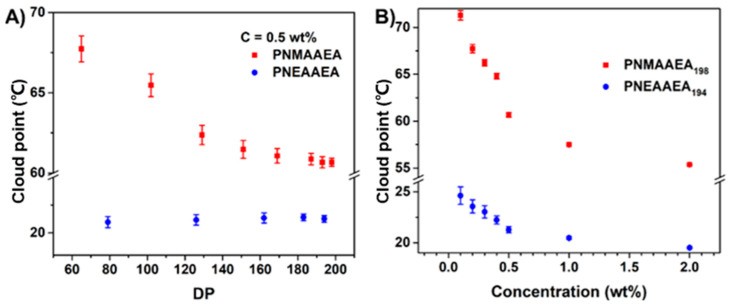
Summaries of the effect of the degree of polymerization (DP) (**A**) and polymer concentration (**B**) on *T*_cp_ of PNMAAEA and PNEAAEA.

It is known that for the typical thermoresponsive PNIPAM, both the polymer concentration and the polymer DP exert almost no or a slight influence on the LCST of PNIPAM, when the DP of PNIPAM is above a given critical point [78]. The thermoresponse of PNMAAEA and PNEAAEA in aqueous solution is shown in Figures S3 and S4, respectively. As summarized in Figure 6, *T*_cp_ of PNEAAEA keeps almost a constant of 20–21 °C, indicating that it is independent of DP in the case of DP above 80 and is independent of polymer concentration (C) in the case of C > 0.5 wt %. However, PNMAAEA is slightly different. Its *T*_cp_ decreases from 67.6 to 61.5 °C when the DP increases from 65 to 151, and *T*_cp_ decreases from 71.3 to 55.4 °C with the increase in polymer concentration from 0.1 to 2.0 wt %, respectively. PNEAAEA and PNMAAEA exhibit different thermoresponsive phenomena, although they have very similar structure, differing in the *N*-alkyl groups of *N*-ethyl and *N*-methyl. The exact reason leading to the different thermoresponse needs further study.

**Figure 7:**
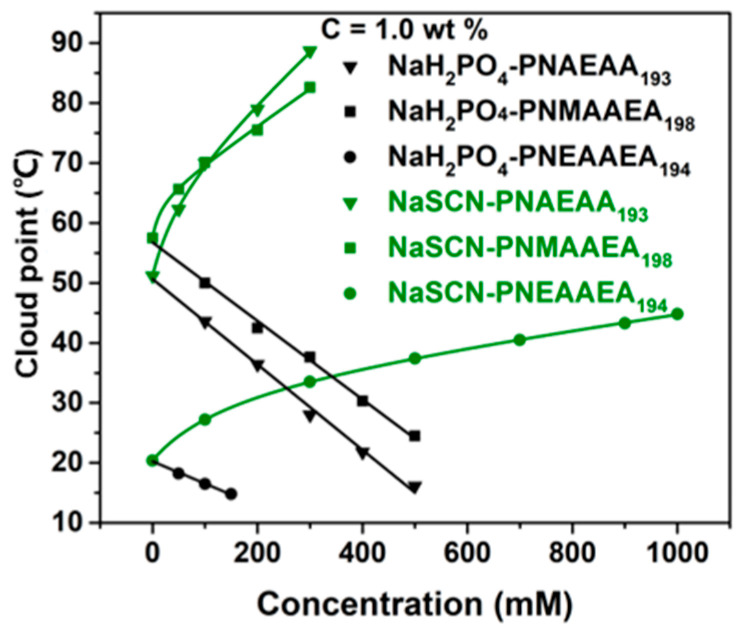
Summaries of the effect of salts on *T*_cp_ of 1.0 wt % aqueous solution of PNAEAA_193_, PNMAAEA_198_, and PNEAAEA_194_.

The influences of inorganic sodium salts on the thermoresponse of PNMAAEA_198_ and PNEAAEA_194_, as well as PNAEAA_193_, are checked. Two typical sodium salts, NaSCN (chaotrope) and NaH_2_PO_4_ (kosmotrope) [22,61,64,72,79,80], are selected. As shown in Figure 7, the *T*_cp_s of the three thermoresponsive homopolymers increase with NaSCN concentration and decrease with NaH_2_PO_4_ concentration. Following the calculations as reported previously [73], Equation (S2) is obtained, from which the *T*_cp_ of the thermoresponsive polymers at a given salt concentration can be determined. The detailed values for the three polymers are summarized in Table S3.

**Figure 8:**
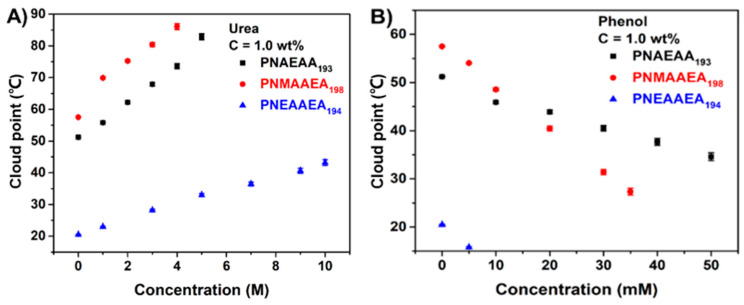
Summaries of the effect of urea (**A**) and phenol (**B**) on *T*_cp_ of 1.0 wt % aqueous solution of PNAEAA_193_, PNMAAEA_198_, and PNEAAEA_194_.

The thermoresponse of PNAEAA, PNMAAEA, and PNEAAEA under different urea and phenol concentrations is shown in Figures S5–S7. As summarized in Figure 8A, urea can increase the *T*_cp_ of the aqueous solution of PNAEAA_193_, PNMAAEA_198_, and PNEAAEA_194_, and their *T*_cp_ increases with urea concentration. It is thought that the urea/poly(2-(*N*-alkylacrylamide) ethyl acetate) complexes formed via hydrogen bonding become more hydrophilic, as discussed previously [73], and therefore, they exhibit a higher *T*_cp_ than that of poly(2-(*N*-alkylacrylamide) ethyl acetate) aqueous solution in the absence of urea. Unlike urea, phenol exerts an opposite effect on *T*_cp_ (Figure 8B), as the phenol/poly(2-(*N*-alkylacrylamide) ethyl acetate) complexes become more hydrophobic.

### 3.3. Synthesis and Thermoresponse of Block Copolymer and Random Copolymer

**Figure 9:**
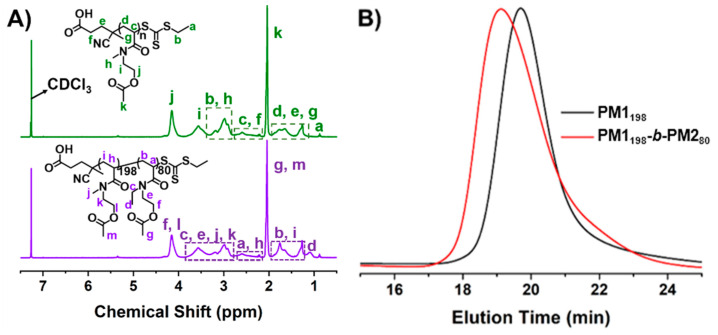
^1^H NMR spectra (**A**) and GPC traces (**B**) of PNMAAEA_198_ and PNMAAEA_198_-*b*-PNEAAEA_80_.

As shown above, PNMAAEA and PNEAAEA have two much different *T*_cp_s. It is thought that the block copolymer may exhibit two separated *T*_cp_s. Following this concern, the PNMAAEA_198_-*b*-PNEAAEA_80_ block copolymer was synthesized employing PNMAAEA_198_ as the macro-CTA. Herein, we employ PNMAAEA but not PNEAAEA, as the macro-CTA is due to the high integrity of CTA moieties in PNMAAEA, as discussed above. Figure 9 shows the ^1^H NMR spectra and GPC traces of PNMAAEA_198_-*b*-PNEAAEA_80_ and its precursor of the PNMAAEA_198_ macro-CTA, and the results are summarized in Table S4.

**Figure 10:**
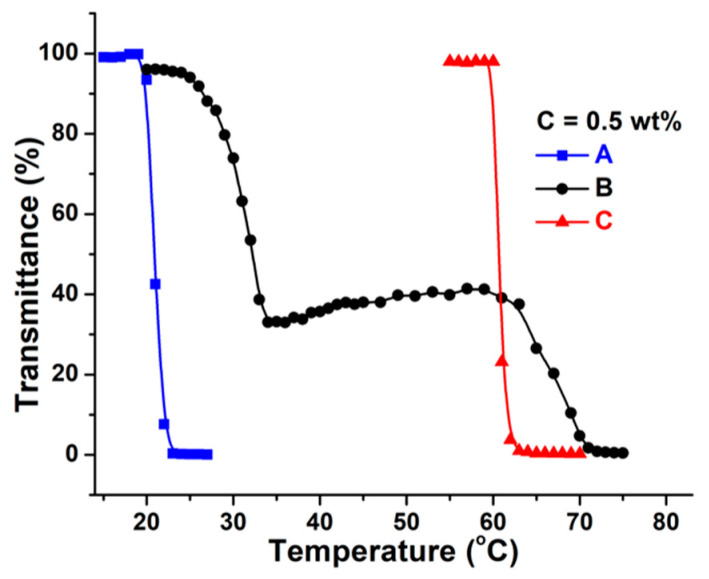
The temperature-dependent transmittance of the 0.5 wt % aqueous solution of PNEAAEA_79_ (**A**), PNMAAEA_198_-*b*-PNEAAEA_80_ (**B**), and PNMAAEA_198_ (**C**).

Figure 10 shows the temperature-dependent transmittance of the aqueous solution of PNMAAEA_198_-*b*-PNEAAEA_80_, as well as the homopolymers of PNEAAEA_79_ and PNMAAEA_198_. As expected, the aqueous solution of PNMAAEA_198_-*b*-PNEAAEA_80_ has two *T*_cp_s, one (30.9 °C, *T*_cp_1) is higher than that of PNEAAEA_79_ (20.9 °C) due to the hydrophilic PNMAAEA_198_ block, and the other (67.7 °C, *T*_cp_2) is slightly higher than that of PNMAAEA_198_ (60.7 °C). The higher *T*_cp_2 may be due to steric repulsion of the PNMAAEA_198_ block tethered on the dehydrated PNEAAEA_80_. It is thought that PNMAAEA_198_-*b*-PNEAAEA_80_ forms colloidal aggregates at temperatures above *T*_cp_1 and *T*_cp_2, which is indicated by the formation of colloidal dispersion, in which the hydrodynamic diameter (*D*_h_) of the colloidal aggregates is confirmed by DLS analysis (Figure S8). Besides, as shown in Figure 10, the soluble-to-insoluble transition of the PNMAAEA_198_-*b*-PNEAAEA_80_ block copolymer at *T*_cp_1 or *T*_cp_2 occurs within a broader temperature window in comparison with those of the corresponding homopolymers. This is due to the hydrophilic PNMAAEA block delaying the soluble-to-insoluble transition of the PNEAAEA block at *T*_cp_1 and the dehydrated PNEAAEA block increasing the steric repulsion of the PNMAAEA block and hindering the soluble-to-insoluble transition of the PNMAAEA block at *T*_cp_2.

**Figure 11:**
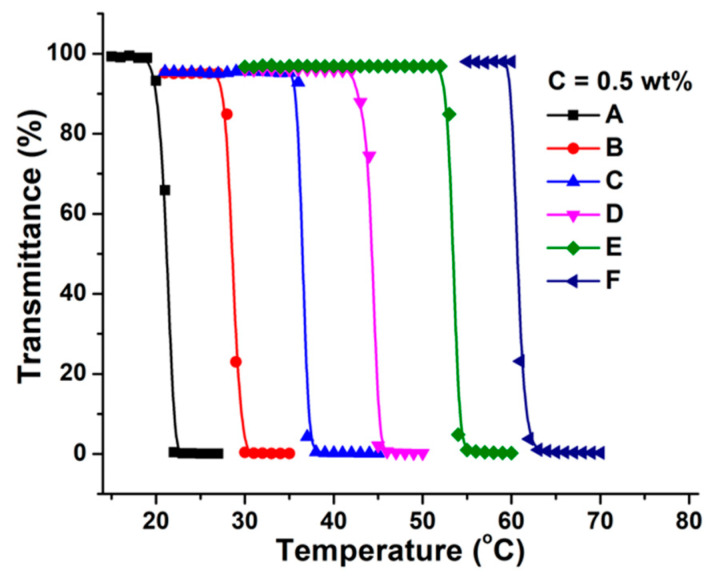
The temperature-dependent transmittance of the 0.5 wt % aqueous solution of PNEAAEA_194_ (**A**), PNMAAEA_50_-*co*-PNEAAEA_150_ (**B**), PNMAAEA_100_-*co*-PNEAAEA_100_ (**C**), PNMAAEA_150_-*co*-PNEAAEA_50_ (**D**), PNMAAEA_175_-*co*-PNEAAEA_25_ (**E**), and PNMAAEA_198_ (**F**).

The much different *T*_cp_s of PNMAAEA and PNEAAEA causes the tuning of *T*_cp_ via synthesis of PNMAAEA-*co*-PNEAAEA random copolymers. These PNMAAEA-*co*-PNEAAEA random copolymers with an almost constant DP at about 200 but different PNMAAEA and/or PNEAAEA fractions were synthesized, and were characterized by ^1^H NMR (Figure S9) and GPC (Figure S10), and the results are summarized in Table S4. As shown in Figure 11, by finely tuning the PNMAAEA and/or PNEAAEA fractions, PNMAAEA-*co*-PNEAAEA random copolymers with a *T*_cp_ ranging from 21.2 to 60.7 °C were obtained. It should be pointed out that PNMAAEA_100_-*co*-PNEAAEA_100_ has a *T*_cp_ of 36.5 °C, very close to human body temperature, which will have potential applications.

## 4. Conclusions

Thermoresponsive poly(2-(*N*-alkylacrylamide) ethyl acetate)s with different *N*-alkyl groups, including PNMAAEA, PNEAAEA, and PNPAAEA, as well as poly(*N*-acetoxylethylacrylamide) (PNAEAA), were synthesized by solution RAFT polymerization. Unexpectedly, it was found that these polymerizations proceed with significant induction periods, and these increase with the length of the *N*-alkyl group in the order of NAEAA < NMAAEA < NEAAEA < NPAAEA. The solubility of poly(2-(*N*-alkylacrylamide) ethyl acetate)s is firmly dependent on the *N*-alkyl groups. PNPAAEA including the largest *N*-propyl group is insoluble in water, whereas PNMAAEA and PNEAAEA are thermoresponsive in water. It is found that the *T*_cp_s of the thermoresponsive polymers with a very similar theoretical DP are in the order of PNEAAEA_194_ < PNAEAA_193_ < PNMAAEA_198_, and PNEAAEA_194_ has a much lower *T*_cp_ than PNMAAEA_198_. The parameters affecting the *T*_cp_ of the thermoresponsive polymers, e.g., DP, polymerization concentration, salt, urea, and phenol, are investigated. The PNMAAEA-*b*-PNEAAEA block copolymer and PNMAAEA-*co*-PNEAAEA random copolymers with different PNMAAEA and/or PNEAAEA fractions were synthesized. Due to the large difference in *T*_cp_s of PNMAAEA and PNEAAEA, the PNMAAEA-*b*-PNEAAEA block copolymer has two separate *T*_cp_s, and the PNMAAEA-*co*-PNEAAEA random copolymers have a *T*_cp_ ranging from 21.2 to 60.7 °C by tuning the PNMAAEA and/or PNEAAEA fractions. Interestingly, PNMAAEA_100_-*co*-PNEAAEA_100_ has a *T*_cp_ of 36.5 °C very close to human body temperature. The thermoresponsive poly(2-(*N*-alkylacrylamide) ethyl acetate)s with tunable thermal response are believed to have potential applications.

## Supplementary Materials

The supplementary materials are available online at https://www.mdpi.com/2073-4360/12/11/2464/s1.
