# Supplementary material for: Thermoresponsive Polymers of Poly(2-(N-alkylacrylamide)ethyl acetate)s
Source: Polymers (Basel). 2020 Oct 24;12(11):2464. doi: 10.3390/polym12112464 (PMC7690893; doi:10.3390/polym12112464)
Supplement: Supplementary file 1 [file polymers-12-02464-s001.pdf]

Supporting information for

Thermoresponsive polymers of poly(2-(*N*-alkylacrylamide) ethyl acetate)s

Xue Liu [1], Yuwen Hou [2], Yimin Zhang [1,*], Wangqing Zhang [2,*]

[1] Key Laboratory for Green Chemical Technology of Ministry of Education, School of Chemical Engineering and Technology, Tianjin University, Tianjin 300350, China; liuxue435@tju.edu.cn

[2] Key Laboratory of Functional Polymer Materials of the Ministry of Education, Institute of Polymer Chemistry, College of Chemistry, Nankai University, Tianjin 300071, China; 1120180337@mail.nankai.edu.cn

* Correspondence: zhangym@tju.edu.cn (Y.Z.); wqzhang@nankai.edu.cn (W.Z.); Tel.: +86-022-23509794 (W.Z.)

1. Chain transfer agent (CTA) of ECT

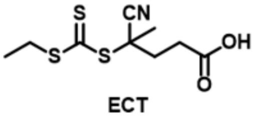

Scheme S1. The RAFT agent of ECT.

2. Summary of the synthesized PNMAAEA, PNEAAEA, PNPAAEA

Table S1 Summaries of the synthesized PNMAAEA, PNEAAEA, PNPAAEA

Entry	monomer	Time (h)	Conv.[a] (%)	DP [b]	M_n (kg/mol^{-1})			$Đ$ [f]
					$M_{n,th}$ [c]	$M_{n,GPC}$ [d]	$M_{n,NMR}$ [e]	
1	NMAAEA	0.75	9.2	18	3.4	3.5	-	1.15
2	NMAAEA	1	26.9	54	9.5	6.6	9.0	1.17
3	NMAAEA	1.5	64.3	129	22.3	13.9	19.9	1.11
4	NMAAEA	2	75.6	151	26.2	16.0	22.4	1.14
5	NMAAEA	3	87.9	176	30.4	17.4	26.0	1.16
6	NMAAEA	4	93.5	187	32.3	18.0	29.1	1.14
7	NMAAEA	5	96.6	193	33.3	18.4	30.1	1.14
8	NMAAEA	6	98.8	198	34.1	18.7	30.3	1.16
9	NEAAEA	1.5	12.8	26	5.0	-	2.6	-
10	NEAAEA	2	39.6	79	14.9	10.7	5.9	1.14
11	NEAAEA	3	62.8	126	23.5	14.1	10.9	1.15
12	NEAAEA	4	80.8	162	30.2	17.3	12.0	1.15
13	NEAAEA	5	91.5	183	34.2	18.2	12.8	1.17
14	NEAAEA	6	97.0	194	36.2	18.9	14.7	1.18
15	NPAAEA	1	3.9	8	1.8	-	-	-
16	NPAAEA	2	14.7	29	6.1	3.5	-	1.39
17	NPAAEA	3	27.1	54	11.1	8.1	-	1.26
18	NPAAEA	4	62.4	125	25.1	14.9	-	1.19
19	NPAAEA	4.5	75.2	150	30.2	16.6	-	1.21
20	NPAAEA	5	87.3	175	35.1	18.0	-	1.22
21	NPAAEA	6	92.4	185	37.1	18.6	-	1.23
22	NPAAEA	7	94.8	190	38.0	19.3	-	1.22

[a] The monomer conversion confirmed by ^{1}H NMR analysis. [b] The polymerization degree (DP) = ([monomer]$_0$/[RAFT]$_0$) × conversion. [c] The theoretical molecular weight according to eqn S1. [d] Molecular weight determined by GPC analysis. [e] Molecular weight determined by ^{1}H NMR analysis. [f] $Đ$ (M_w/M_n) values determined by GPC analysis.

The formula for calculating theoretical molecular weight:

$$M_{n,th} = \frac{[\text{monomer}]_0 \times M_{\text{monomer}}}{[\text{RAFT}]_0} \times \text{conversion} + M_{\text{RAFT}} \qquad \text{(S1)}$$

Note: $[\text{monomer}]_0$ and $[\text{RAFT}]_0$ represent the number of moles of monomer and RAFT we added at the beginning of the reaction, respectively. The conversion is confirmed by comparing the integral areas of the monomer protons of C=C–*H* at δ = 5.49-5.52, 5.54-5.57, 5.48-5.51 ppm for NMAAEA, NEAAEA, NPAAEA respectively, with those of the 1,3,5-trioxane internal standard at δ = 5.15 ppm in ^{1}H NMR spectra.

3. The ^{1}H NMR spectra of the NMAAEA, NEAAEA, NPAAEA

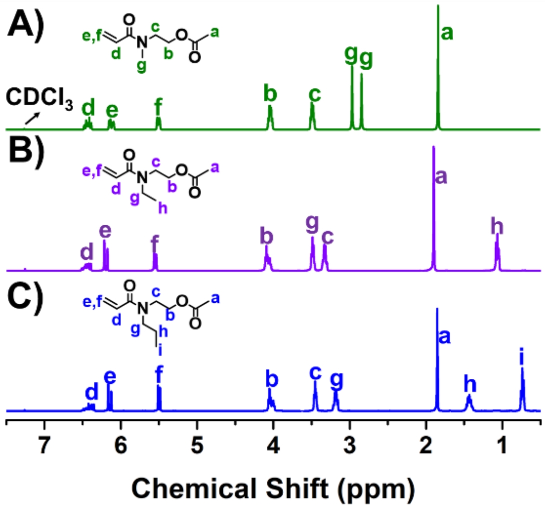

Fig. S1 The ^{1}H NMR spectra of NMAAEA (A)**,** NEAAEA (B), NPAAEA (C) in $CDCl_3$.

4. Kinetics of solution RAFT polymerization

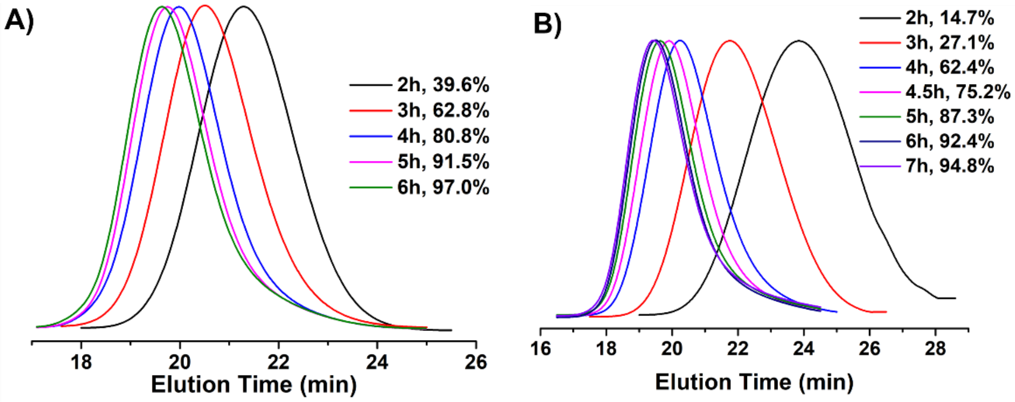

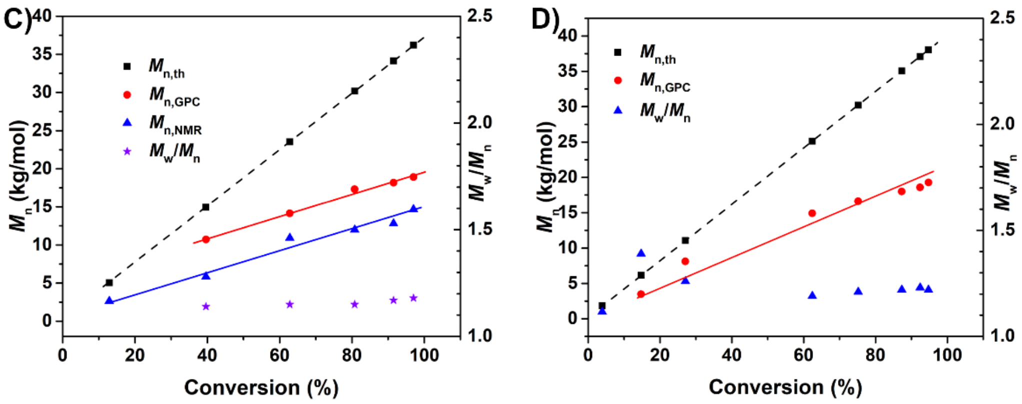

Fig. S2 Kinetics of solution RAFT polymerization of NEAAEA and NPAAEA. The GPC traces of PNEAAEA (A), the GPC traces of PNPAAEA (B), molecular weight and the *Đ* (M_w/M_n) value of PNEAAEA at different conversion(C), molecular weight and the *Đ* (M_w/M_n) value of PNPAAEA at different conversion(D). Polymerization conditions: $[\text{Monomer}]_0 : [\text{RAFT}]_0 : [\text{AIBN}]_0$ = 1000:5:1, 70°C, solid content: 33.3%, solvent: DMF.

5. Summary of the T_{cp} of PNAEAA, PNMAAEA, PNEAAEA

Table S2. Summary of the T_{cp} of PNAEAA, PNMAAEA ,PNEAAEA

Polymer	T_{cp} determined at 50% change of the transmittance (°C)	T_{cp} determined at the inflection points (°C)
$PNAEAA_{193}$	50.4	48.8
$PNMAAEA_{198}$	57.5	56.4
$PNEAAEA_{194}$	20.5	19.4
$PNMAAEA_{198}$-*b*-$PNEAAEA_{80}$	30.9 ($T_{cp}1$)<br>67.7 ($T_{cp}2$)	24.2 ($T_{cp}1$)<br>61.3 ($T_{cp}2$)

6. Thermoresponse of PNMAAEA, PNEAAEA in aqueous solution

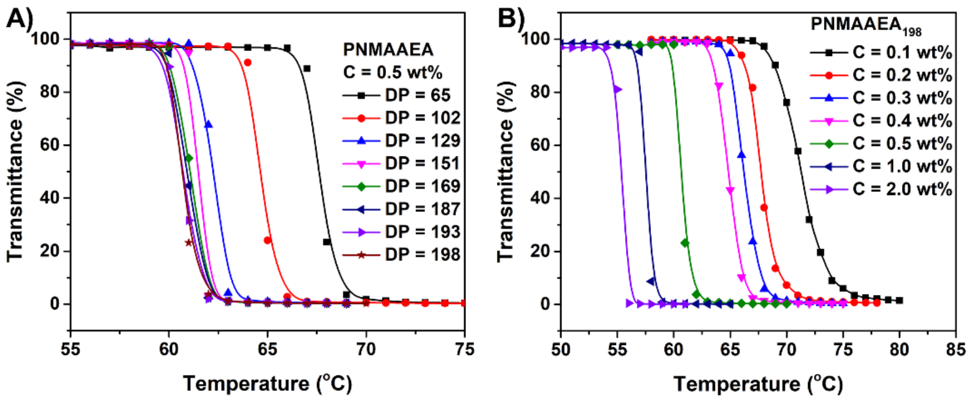

Fig.S3 Temperature-dependent transmittance of 0.5 wt% aqueous solution of PNMAAEA with different DPs (A) and temperature-dependent transmittance of the $PNMAAEA_{198}$ aqueous solution with the polymer concentration ranging from 0.1 wt% to 2.0 wt% (B).

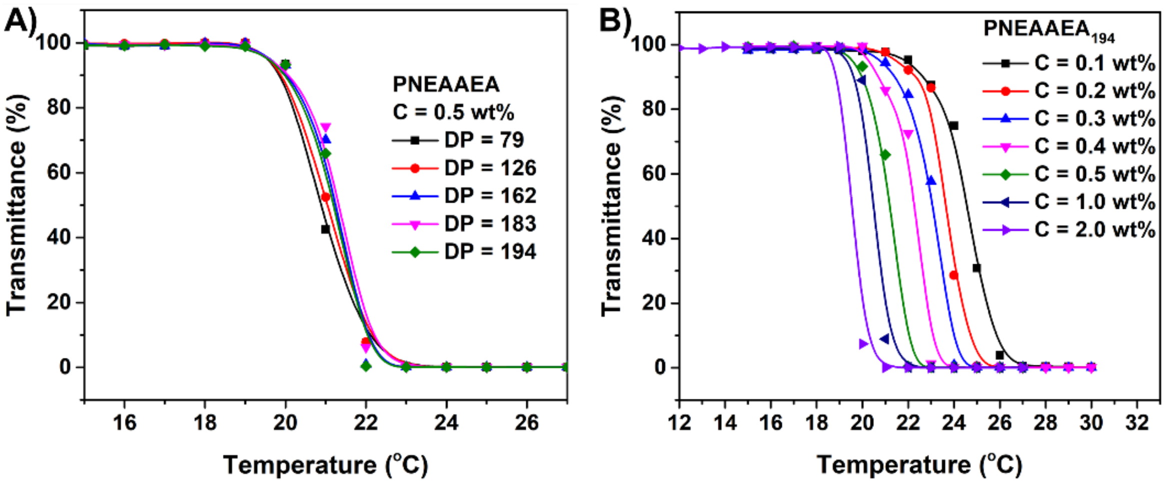

Fig.S4 Temperature-dependent transmittance of 0.5 wt% aqueous solution of PNEAAEA with different DPs (A) and temperature-dependent transmittance of the $PNEAAEA_{194}$ aqueous solution with the polymer concentration ranging from 0.1 wt% to 2.0 wt% (B).

7. Calculation results of the corresponding parameters for salt effect

Table S3. Fitted values for c, B_{max} and K_d from the T_{cp} data of $PNAEAA_{193}$, $PNMAAEA_{198}$, $PNEAAEA_{190}$ with two sodium salts.

Na^+ Counter antion	polymer	c (°C/mol)	B_{max} (°C)	K_d (M)
	$PNAEAA_{193}$	78.65	16.42	0.06
SCN^-	$PNMAAEA_{198}$	60.65	7.02	0.02
	$PNEAAEA_{194}$	11.63	14.75	0.16
	$PNAEAA_{193}$	-71.23	0	0
$H_2PO_4^-$	$PNMAAEA_{198}$	-65.43	0	0
	$PNEAAEA_{194}$	-37.00	0	0

$$T_{cp}\,(^{o}C) = T_0 + c[M] + \frac{B_{max}[M]}{K_d + [M]} \qquad (S2)$$

in which T_0 is the T_{cp} of 1.0 wt% polymer aqueous solution in absence of any salt, c is a constant on behalf of the slope of the line with the units of temperature/concentration (℃/[M]) and is relevant to the salting-out efficacy of a specific salt, K_d is unitless and represent the apparent equilibrium dissociation constant of the anion-polymer interaction, B_{max} represents the difference between T_{cp} at salt saturation concentration and T_0, respectively. The detailed values are summarized in Table S3.

8. Thermoresponse of PNAEAA, PNMAAEA, PNEAAEA under different urea and phenol concentrations

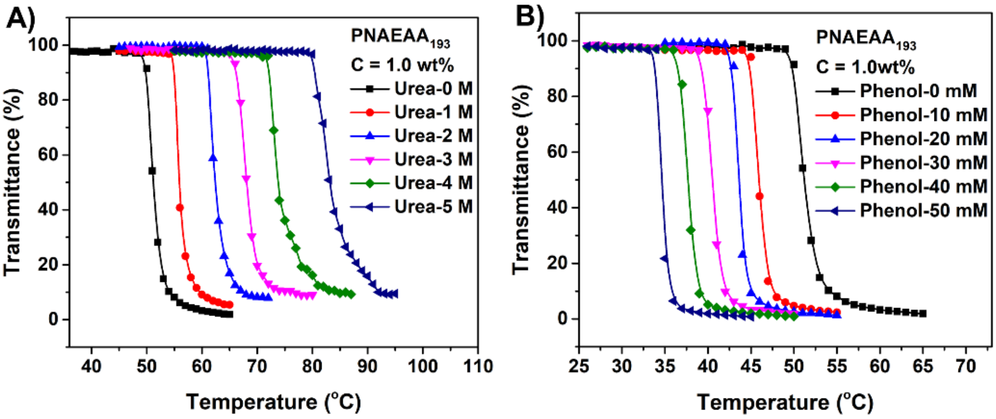

Fig.S5 Temperature-dependent transmittance of 1.0 wt% $PNAEAA_{193}$ aqueous solution in presence of different concentration of urea (A) and phenol (B).

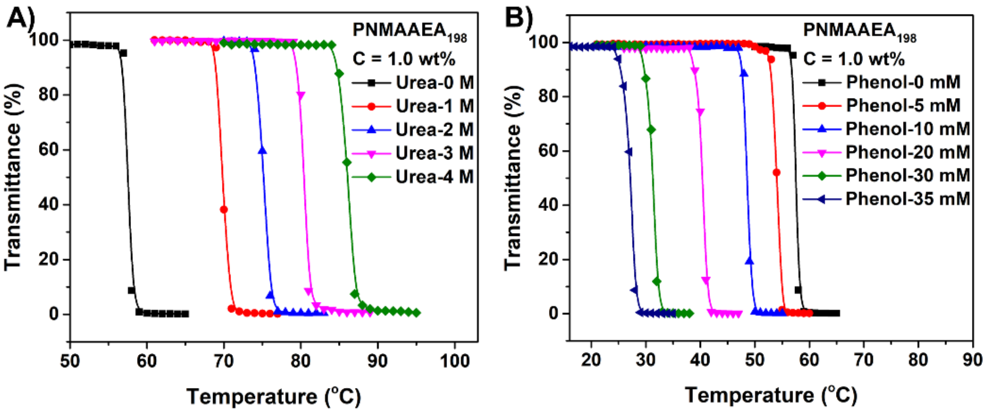

Fig.S6 Temperature-dependent transmittance of 1.0 wt% $PNMAAEA_{198}$ aqueous solution in presence of different concentration of urea (A) and phenol (B).

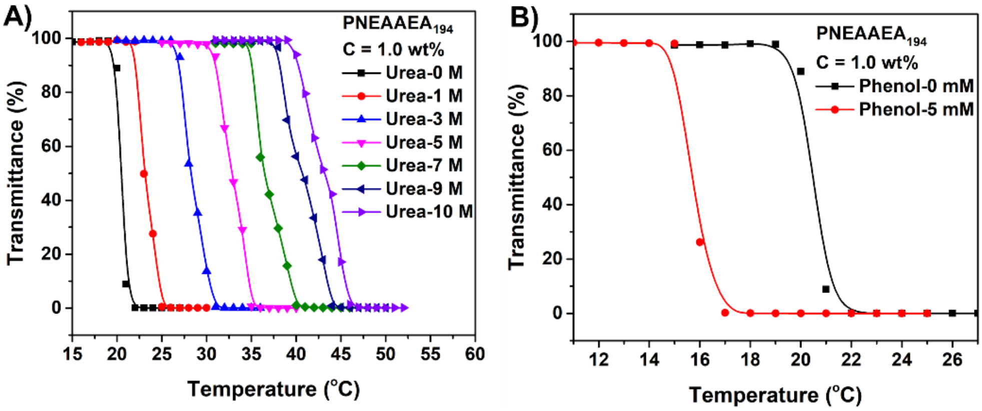

Fig.S7 Temperature-dependent transmittance of 1.0 wt% $PNEAAEA_{194}$ aqueous solution in presence of different concentration of urea (A) and phenol (B).

9. The summary of the synthesized block/random copolymer and the corresponding homopolymer

Table S4 The molecular weight and $Đ$ (M_w/M_n) of the synthesized block/random copolymer and the corresponding homopolymer.

Entry	polymer	M_n (kg/mol^{-1})		$Đ$ ^{c}
		$M_{n,th}$ ^{a}	$M_{n,GPC}$ ^{b}	
1	PNMAAEA$_{198}$	34.30	18.27	1.16
2	PNMAAEA$_{198}$-*b*-PNEAAEA$_{80}$	49.15	19.79	1.25
3	PNMAAEA$_{50}$-*co*-PNEAAEA$_{150}$	36.61	20.77	1.15
4	PNMAAEA$_{100}$-*co*-PNEAAEA$_{100}$	36.90	20.90	1.15
5	PNMAAEA$_{150}$-*co*-PNEAAEA$_{50}$	35.20	19.85	1.38
6	PNMAAEA$_{175}$-*co*-PNEAAEA$_{25}$	34.85	17.33	1.16

^{a} The theoretical molecular weight according to eqn (S1). ^{b} Molecular weight determined by GPC analysis. ^{c} The $Đ$ (M_w/M_n) values determined by GPC analysis.

10. The dynamic light scattering (DLS) analysis of PNMAAEA$_{198}$-*b*-PNEAAEA$_{80}$

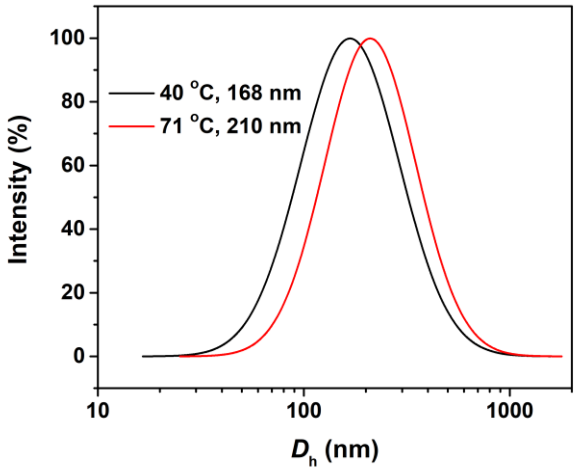

Fig.S8 Hydrodynamic diameter (D_h) distribution of 0.5 wt% aqueous solution of PNMAAEA$_{198}$-*b*-PNEAAEA$_{80}$ block copolymer at 40 °C and 71 °C.

11. The ^{1}H NMR spectra and the GPC traces of PNMAAEA-*co*-PNEAAEA

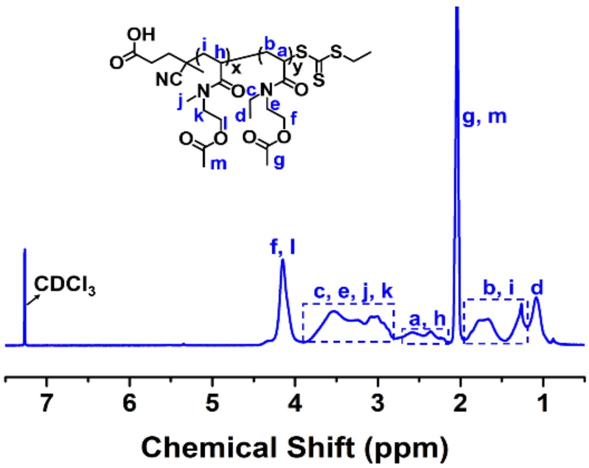

Fig.S9 ^{1}H NMR spectra of PNMAAEA$_{100}$-*co*-PNEAAEA$_{100}$ in $CDCl_3$.

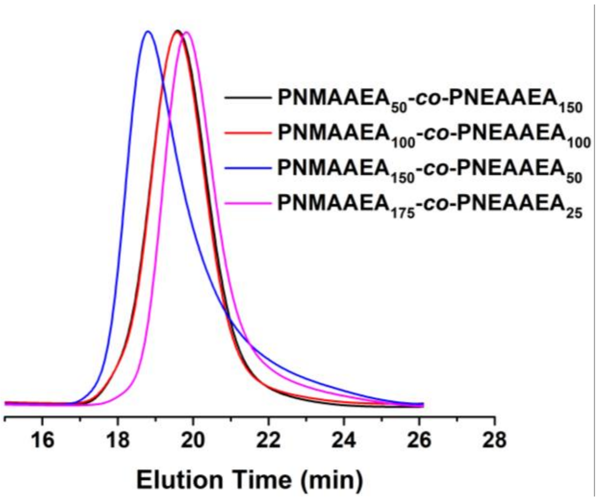

Fig.S10 GPC traces of PNMAAEA-*co*-PNEAAEA**.**
